# Autochthonous *Leishmania* (*Viannia*) *lainsoni* in Dog, Rio de Janeiro State, Brazil, 2023

**DOI:** 10.3201/eid3105.241058

**Published:** 2025-05

**Authors:** Isabela Cordeiro da Silva Santos, Daniel Moreira de Avelar, Luciana de Freitas Campos Miranda, Artur Augusto Velho Mendes Júnior, Lucas Keidel Oliveira, Luanna da Silva Ventura, Aline Fagundes da Silva, Fernanda Nunes Santos, Liliane de Fátima Antônio Oliveira, Rodrigo Caldas Menezes, Andreza Pain Marcelino

**Affiliations:** Fundação Oswaldo Cruz, Rio de Janeiro, Brazil (I.C.d.S. Santos, L.d.F.C. Miranda, L.K. Oliveira, L.d.S. Ventura, A.F. da Silva, F.N. Santos, L.d.F.A. Oliveira, R.C. Menezes, A.P. Marcelino); Fundação Oswaldo Cruz, Belo Horizonte, Brazil (D.M. de Avelar); Instituto Carlos Chagas, Curitiba, Brazil (A.A.V.M. Júnior)

**Keywords:** visceral leishmaniasis, vector-borne infections, parasites, *Leishmania*, dog, Brazil

## Abstract

In Brazil, *Leishmania* (*Leishmania*) *infantum* causes canine visceral leishmaniasis; the primary vector is the *Lutzomyia longipalpis* sand fly. We describe a case of canine visceral leishmaniasis caused by *Leishmania (Viannia) lainsoni* in a dog from Barra Mansa municipality, Rio de Janeiro state. Better specificity of serologic diagnostic techniques is needed for diagnoses.

Protozoa transmitted by sand flies cause leishmaniasis, and several pathogenic species affect humans. Various clinical forms of the disease have been described, including visceral, cutaneous, and mucocutaneous leishmaniasis (*1*). *Leishmania* (*Viannia*) *lainsoni* was described in Brazil in 1987 as the causative agent of human cases of cutaneous leishmaniasis. Its vector is the *Lutzomyia ubiquitalis* sand fly (*2*). Other countries in Latin America have reported human cases of *L*. (*V*.) *lainsoni* infection. Researchers isolated the parasite from the rodent species *Cuniculus paca*, the lowland paca, in the state of Pará, Brazil, suggesting a potential wild reservoir (*3*,*4*).

This study reports the case of a dog (*Canis familiaris*) infected with *L*. (*V*.) *lainsoni* that was from the municipality of Barra Mansa, an urban area in Rio de Janeiro state with widespread visceral leishmaniasis (VL) (Appendix). The Ethics Committee on the Use of Animals–Fiocruz approved this work (license no. LW 19/20; https://www.ceua.fiocruz.br/ceuaw000.aspx).

A 5-year-old male dog of mixed breed domiciled in Barra Mansa tested positive for VL by both rapid immunochromatographic testing and enzyme immunoassay (Bio-Manguinhos, https://portal.fiocruz.br/en/unidade/immunobiological-technology-institute-biomanguinhos) during epidemiologic surveillance in 2023 and was euthanized using the recommendations of the Brazilian Ministry of Health (https://www.gov.br). The dog had not moved to other regions and had localized alopecia, crusted skin ulcers, onychogryphosis, keratoconjunctivitis, normocytic normochromic anemia, hyperproteinemia, hyperglobulinemia, hypoalbuminemia, and a low albumin:globulin ratio (Appendix). Histopathologic changes included skin with hyperkeratosis and multifocal and moderate granulomatous dermatitis, as well as lymphoid hyperplasia of the spleen. Immunohistochemistry was positive for amastigote forms of *Leishmania* in skin and spleen (Figure 1).

**Figure 1:**
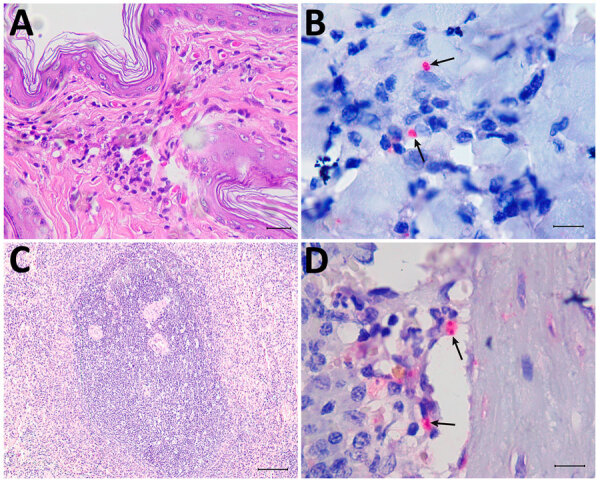
Histologic sections from autochthonous *Leishmania* (*Viannia*) *lainsoni* in dog (*Canis familiaris*), Rio de Janeiro state, Brazil, 2023. A, B) Skin of the examined dog: hyperkeratosis and moderate granulomatous infiltrate in the dermis are composed mainly of macrophages, with a smaller number of plasma cells and lymphocytes (A) and red-stained amastigotes in the cytoplasm of macrophages (arrows) (B). C, D) Spleen of the examined dog: lymphoid hyperplasia (C) and red-stained amastigotes in the cytoplasm of macrophages in the parenchyma (arrows) (D). A, C) Hematoxylin-eosin stain; B, D) immunohistochemistry. Scale bars indicate 10 µm.

We performed parasitologic and PCR tests (Appendix Table 2). We used multilocus enzyme electrophoresis with 5 enzyme profiles (phosphoglucomutase, glucose-6-phosphate dehydrogenase, nucleoside hydrolase, 6-phosphogluconate dehydrogenase, and phosphoglucose isomerase) (*5*). We extracted DNA from the isolated parasite and used it for PCR restriction fragment length polymorphism analysis (*Hae*III and *Bst*UI). We sequenced the 70-kDa heat shock protein products with the same primers by Sanger sequencing (primers: BankIt2825220 and Seq1PP760383) (*6*). Those techniques identified the parasite as *L*. (*V*.) *lainsoni* in all profiles studied in the bone marrow sample (Figure 2).

**Figure 2:**
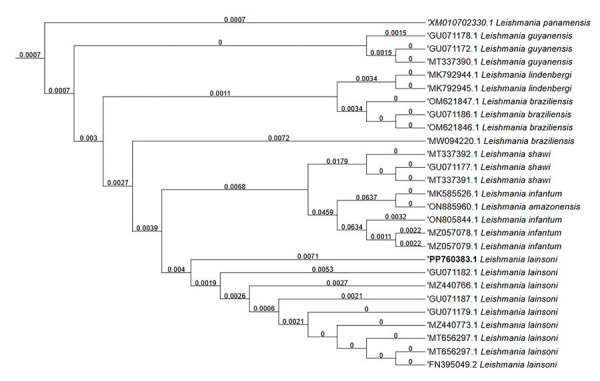
Evolutionary analysis of autochthonous *Leishmania* (*Viannia*) *lainsoni* in dog (*Canis familiaris*), Rio de Janeiro state, Brazil, 2023. Bold text indicates isolate from this study. Evolutionary history was inferred by using the maximum-likelihood method and Kimura 2-parameter model. The bootstrap consensus tree inferred from 1,000 replicates represents evolutionary history of the taxa analyzed. Branches corresponding to partitions reproduced in <50% bootstrap replicates are collapsed. The percentage of replicate trees in which the associated taxa clustered together in the bootstrap test (1,000 replicates) are shown next to the branches. Initial tree(s) for the heuristic search were obtained by applying the BioNJ neighbor-joining method to a matrix of pairwise distances estimated by using the maximum composite likelihood approach. This analysis involved 33 nucleotide sequences. There were a total of 463 positions in the final dataset. Evolutionary analyses were conducted in MEGA11 (https://www.megasoftware.net). MEGA used the first position for each codon in the construction of the phylogenetic tree. GenBank accession numbers are shown.

Like other species of the subgenus *Viannia*, *L*. (*V*.) *lainsoni* can cause ulcerative or nodular dermal lesions in humans (*4*). The clinical signs found in this infected dog included onychogryphosis and skin alterations. Development of skin lesions can lead to hematogenous dissemination and parasitemia of internal organs, as observed in this case, and visceral involvement of lymph nodes and spleen (*7*). The positive results of serologic tests show flaws in the specificity of the techniques because those tests were validated for detecting dogs with canine VL caused by *L*. (*Leishmania*) *infantum*. Hematocrit values less than the reference range, along with a slight increase in total protein, are expected in chronic diseases. We observed no changes in renal function markers. The host–parasite interaction has been extensively studied in dogs infected with *L*. (*L*.) *infantum*; however, little is known about that interaction in dogs infected with other *Leishmania* species.

In Latin America, *L*. (*V*.) *lainsoni* is found in tropical and sub-Andean regions with different climatic conditions. Its presence in other countries highlights the high dispersal capacity of the parasite and potential involvement of unidentified mammalian host vectors. Barra Mansa has a crucial migratory flow because it is located on the banks of the Paraíba do Sul River and influences the Médio Paraíba region and southern part of the south-central region of Rio de Janeiro State.

The dog lived in an area surrounded by natural and abundantly wooded areas. A large portion of the Hemlock Forest is located in Barra Mansa, and *C. paca* rodents are part of the local fauna and could serve as reservoirs of *L*. (*V*.) *lainsoni* in that area (*8*).

Few entomologic surveys have been conducted in Barra Mansa, and only *Lu*. *sallesi* and *Lu*. *longipalpis* sand flies were confirmed, limiting the conclusions of this study (*9*). Although the *Lu. ubiquitalis* sand fly is considered the primary vector of *L*. (*V*.) *lainsoni* in Brazil, other species such as *Lu*. *nuneztovari anglesi* and *Lu*. *velascoi* sand flies in Bolivia have been reported (*10*). Therefore, identification of a dog infected with *L*. (*V*.) *lainsoni* in Barra Mansa may be linked to transmission by other yet undocumented sand fly species in that municipality. The dog did not have a history of moving to other locations. We consider environmental changes caused by humans in the region, as well as local wildlife and migratory flows, as possible causes of infection. This study raises several questions. Is a new and yet unknown disease cycle being established locally? What is the risk for the disease becoming endemic in the population? Will the cycle persist? Also, could other regions in Brazil or elsewhere face similar risks of emerging *Leishmania* species infecting dogs? Further epidemiologic investigations and taxonomic characterization studies are essential and should be continuously supported. Efforts to create clearer specificity in serologic diagnostic techniques are also needed.

Appendix Additional information for autochthonous *Leishmania* (*Viannia*) *lainsoni* in dog, Rio de Janeiro state, Brazil, 2023
