# Supplementary material for: Autochthonous Leishmania (Viannia) lainsoni in Dog, Rio de Janeiro State, Brazil, 2023
Source: Emerg Infect Dis. 2025 May;31(5):1039–42. doi: 10.3201/eid3105.241058 (PMC12044231; doi:10.3201/eid3105.241058)
Supplement: Appendix — Additional information for autochthonous Leishmania (Viannia) lainsoni in dog, Rio de Janeiro state, Brazil, 2023 [file 24-1058-Techapp-s1.pdf]

Article DOI: https://doi.org/10.3201/eid3105.241058

EID cannot ensure accessibility for supplementary materials supplied by authors. Readers who have difficulty accessing supplementary content should contact the authors for assistance.

Autochthonous *Leishmania* (*Viannia*) *lainsoni* in Dog, Rio de Janeiro State, Brazil, 2023

Appendix

Appendix Table 1. Hematologic and biochemical profile of a dog with leishmaniasis Barra Mansa-Rio de Janeiro, 2023*

Profile	Result	Result compared with referent	Referent
Complete blood count			
Erythrocytes	4.28	↓	5.50–8.50 million/µL
Hemoglobin	9.30	↓	12.00–18.00 g/dL
Hematocrit	27.1	↓	37.00%–55.00%
MCV	63.3		60,00–77,00 fL
HCCM	34.31		31.00–36.00 g/dL
RDW-CV	14.8		12.00%–15.00%
Platelets	150,000/µL		150,000–500,000/µL
Leukogram			
Leukocytes	12710/µL		5,500–16,500/µL
Basophils	0		0–495/µL
Eosinophils	127/µL		0–1650/µL
Myelocytes	0		0/µL
Metamyelocyte	0		0/µL
Rods	0		0–495/µL
Segmented	7,753/µL		3,300–12,705/µL
Lymphocytes	3,305/µL		660–4,950/µL
Monocytes	1,525/µL		0–1,650/µL
Biochemistry			
Total plasma protein	9.00 g/dL	↑	6.0–8.0 g/dL
Creatine	0.9 mg/dL		0.50–1.50 mg/dL
GGT	2.0 U/L		1.0–10.0 U/L
ALT/TGP	17.44 U/L		10.0–102.0 U/L
AST/TGO	39.90 U/L		10.0–88.0 U/L
Alkaline phosphatase	15.68 U/L		20.0–156.0 U/L
Urea	57.15 mg/dL		12.0–60.0 mg/dL
Total protein	7.84 g/dL		5.40–7.80 g/dL
Albumin	2.28 g/dL		2.50–4.20 g/dL
Globulin	5.56 g/dL	↑	2.30–5.20 g/dL
Albumin/globulin ratio	0.41	↓	0.60–1.50
Total bilirubin	0.27 mg/dL		0.10–0.60 mg/dL
Direct bilirubin	0.06 mg/dL		0.05–0.30 mg/dL
Indirect bilirubin	0.21 mg/dL		0.01–0.40 mg/dL

*Sources: Kaneko JJ et al. Clinical biochemistry of domestic animals, 6th ed. New York: Academic; 2008. Cornell University, Animal Health Diagnostic Center, Ithaca, New York, USA, 2016. ↑, increase; ↓, decrease.

Appendix Table 2. Results of the parasitologic and PCR tests performed on a dog with leishmaniasis from Barra Mansa, Rio de Janeiro State, 2023

Clinical sample	Cytology	Culture	PCR/HSP70–240 bp
Spleen	Positive	Negative	Negative
Popliteal lymph node tissue	*	*	*
Bone marrow aspirate	Negative	Positive	Positive
Popliteal lymph node aspirate	Negative	Negative	Negative
Skin	Negative	Negative	Negative

*Not performed.

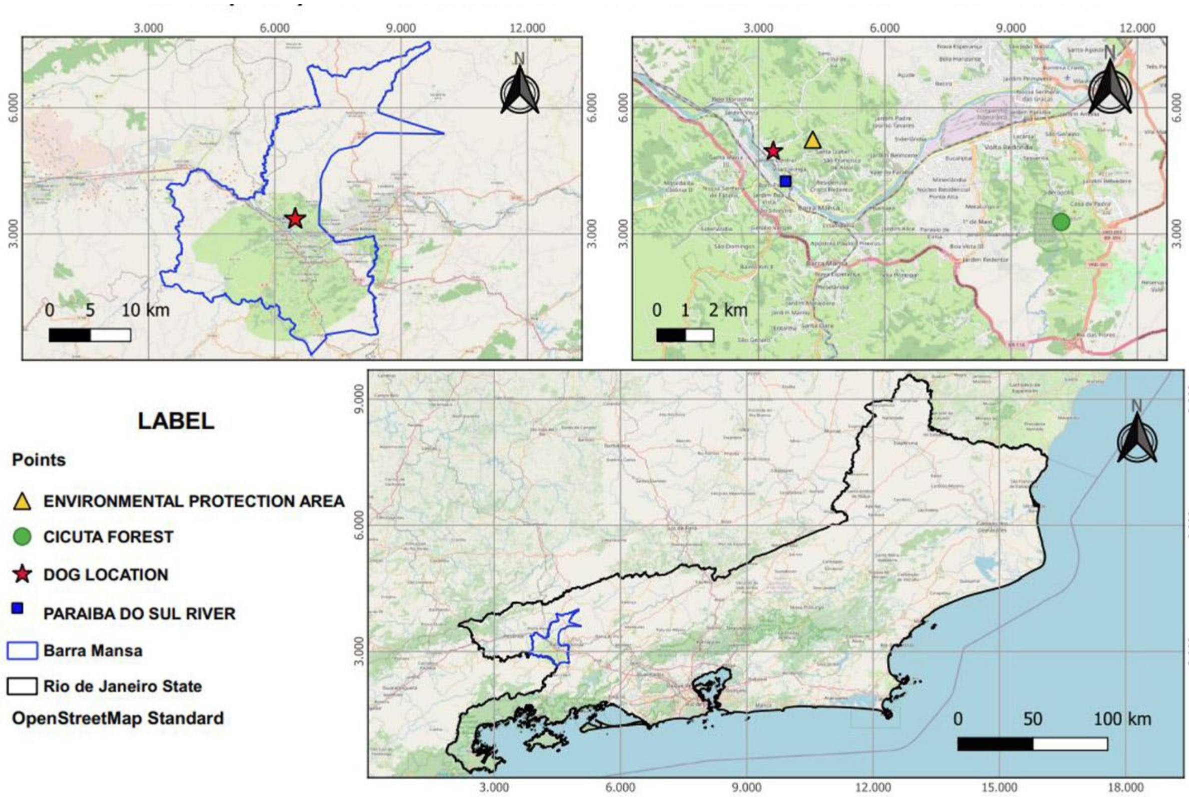

Appendix Figure 1. Maps of Rio de Janeiro State, the municipality of Barra Mansa, and environmental areas close to the dog's address (highlighted): Candido Silva Environmental Protection Area, Cicuta Forest, and Paraíba do Sul River. This map was created using QGIS 3.28.9 software and cartographic base of Rio de Janeiro State obtained from the Brazilian Institute of Geography and Statistics (https://ibge.gov.br/).

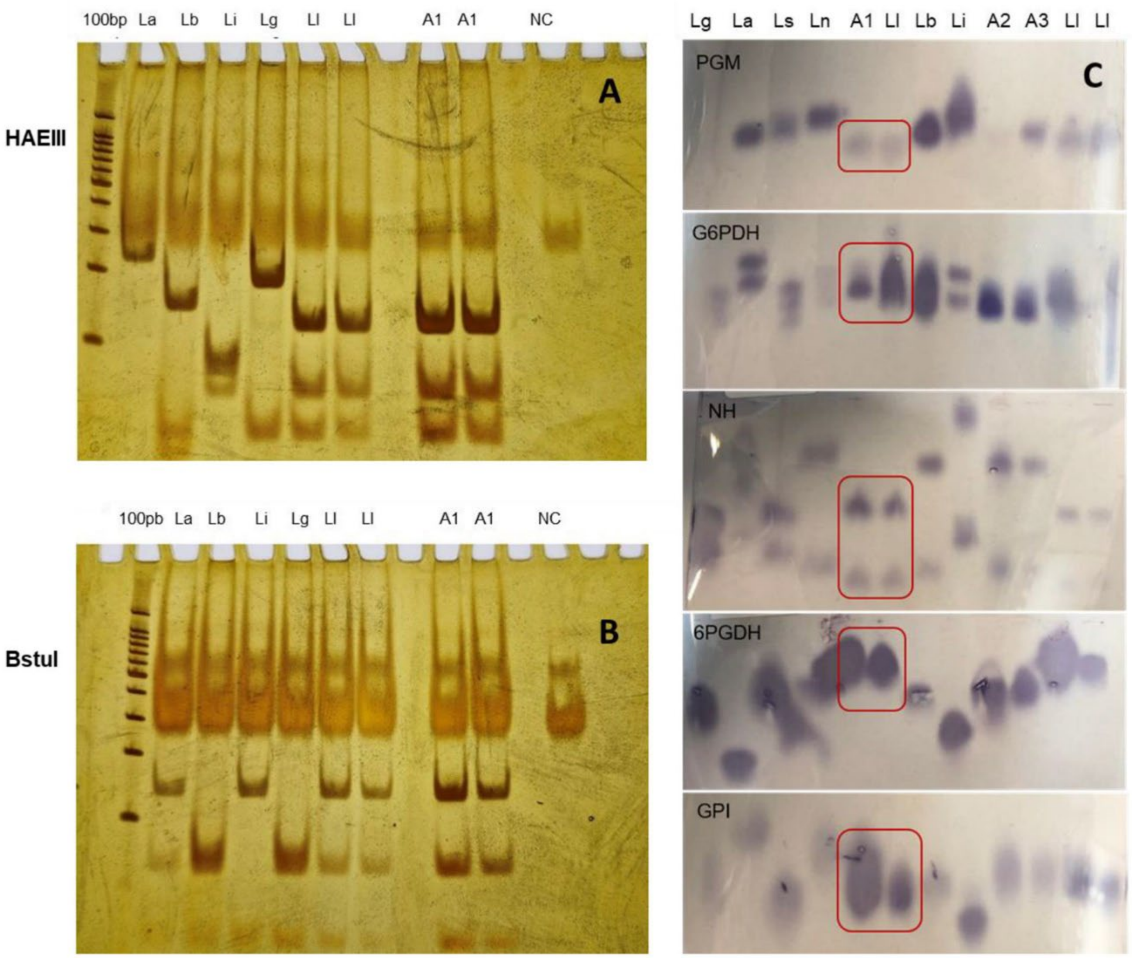

Appendix Figure 2. PCR-RFLP 234-bp heat-shock protein (HSP70) showing the (A) *Hae*III and (B) *Bst*UI profiles of the dog infected with *Leishmania lainsoni* compared with profiles of reference strains. C) Isozyme electrophoresis showing the enzyme profiles of the dog for *L. lainsoni* compared with reference strains and human patients. The figure shows the 5 enzymes studied. The order of parasites from left to right is La, Ls, Ln, A1, Ll, Lb, and A2 and A3. A1, dog with *Leishmania lainsoni*; A2 and A3, patients with *Leishmania braziliensis;* bp, 100-bp molecular weight standard; La, *Leishmania amazonensis*/IFLA/BR/1967/PH-8; Lb, *Leishmania braziliensis*/MHOM/BR/75/M2903; Lg, *Leishmania guyanensis*/MHOM/BR/1975/M4147; Li, *Leishmania infantum*/MHOM/BR/74/PP75; Ll, *Leishmania lainsoni*/MHOM/BR/81/M6426; Ln, *Leishmania naiffi*/MDAS/BR/1979/M5533; Ls, *Leishmania shawi*/ MCEB/BR/1984/M8408; NC, negative control.
